# Usability of Three-dimensional Augmented Visual Cues Delivered by Smart Glasses on (Freezing of) Gait in Parkinson’s Disease

**DOI:** 10.3389/fneur.2017.00279

**Published:** 2017-06-13

**Authors:** Sabine Janssen, Benjamin Bolte, Jorik Nonnekes, Marian Bittner, Bastiaan R. Bloem, Tjitske Heida, Yan Zhao, Richard J. A. van Wezel

**Affiliations:** ^1^Biomedical Signal and Systems Group, MIRA Institute for Biomedical Technology and Technical Medicine, University of Twente, Enschede, Netherlands; ^2^Department of Neurology, Donders Institute for Brain, Cognition and Behavior, Radboud University Medical Center, Nijmegen, Netherlands; ^3^Department of Rehabilitation, Donders Institute for Brain, Cognition and Behavior, Radboud University Medical Center, Nijmegen, Netherlands; ^4^Department of Biophysics, Donders Institute of Brain, Cognition and Behavior, Radboud University, Nijmegen, Netherlands

**Keywords:** Parkinson’s disease, freezing of gait, wearables, smart glasses, augmented reality, external cueing, visual cues

## Abstract

External cueing is a potentially effective strategy to reduce freezing of gait (FOG) in persons with Parkinson’s disease (PD). Case reports suggest that three-dimensional (3D) cues might be more effective in reducing FOG than two-dimensional cues. We investigate the usability of 3D augmented reality visual cues delivered by smart glasses in comparison to conventional 3D transverse bars on the floor and auditory cueing *via* a metronome in reducing FOG and improving gait parameters. In laboratory experiments, 25 persons with PD and FOG performed walking tasks while wearing custom-made smart glasses under five conditions, at the end-of-dose. For two conditions, augmented visual cues (bars/staircase) were displayed *via* the smart glasses. The control conditions involved conventional 3D transverse bars on the floor, auditory cueing *via* a metronome, and no cueing. The number of FOG episodes and percentage of time spent on FOG were rated from video recordings. The stride length and its variability, cycle time and its variability, cadence, and speed were calculated from motion data collected with a motion capture suit equipped with 17 inertial measurement units. A total of 300 FOG episodes occurred in 19 out of 25 participants. There were no statistically significant differences in number of FOG episodes and percentage of time spent on FOG across the five conditions. The conventional bars increased stride length, cycle time, and stride length variability, while decreasing cadence and speed. No effects for the other conditions were found. Participants preferred the metronome most, and the augmented staircase least. They suggested to improve the comfort, esthetics, usability, field of view, and stability of the smart glasses on the head and to reduce their weight and size. In their current form, augmented visual cues delivered by smart glasses are not beneficial for persons with PD and FOG. This could be attributable to distraction, blockage of visual feedback, insufficient familiarization with the smart glasses, or display of the visual cues in the central rather than peripheral visual field. Future smart glasses are required to be more lightweight, comfortable, and user friendly to avoid distraction and blockage of sensory feedback, thus increasing usability.

## Introduction

In advanced disease stages, most persons with Parkinson’s disease (PD) experience freezing of gait (FOG): sudden paroxysmal gait arrests preventing effective forward movement (1, 2). FOG negatively impacts mobility and independence and is associated with falls, fall-related injuries, and emotional stress in social situations, resulting in a reduced quality of life (3, 4). Tight turns, narrow passages, gait initiation, and approaching a destination are well-known triggers for FOG (1). Apart from episodes of FOG, persons with PD and FOG (PD-FOG) display continuous gait abnormalities such as increased stride variability (5).

External cues (i.e., transverse bars on the floor or walking at the rhythm of a metronome) are well-known strategies to reduce FOG (6) and improve speed, cadence (7–9), and stride length variability (10–12), with an additional increase in step length for visual cues (7–9). Despite their potential effectiveness, the use of cues is limited by practical constraints such as a lack of portability and hindrance of bystanders (e.g., housemates). Smart glasses, also called augmented reality (AR) glasses, have the potential to provide portable, personalized cues in an AR overlay on top of a user’s visual field. Smart glasses have been welcomed as an assistive technology to facilitate daily living by a majority of respondents in a user requirement survey amongst persons with PD (13, 14).

A previous study compared the effects of rhythmic flashes, a visual flow, and a static placebo delivered by virtual reality glasses with transverse lines on the floor on FOG and gait parameters. This study found a deterioration of gait with rhythmic flashes, a marginal improvement only of task completion time with the virtual visual flow, and the largest improvement of FOG and gait parameters with transverse lines on the floor (15). In another study, three types of external cues (a metronome, flashing light, and optic flow) delivered *via* the Google Glass reduced the variability of cadence and stride length, suggesting a more stable gait pattern (12). There was no significant effect on FOG, possibly due to a low overall number of FOG episodes. Some participants disliked the placement of the display in the right upper corner, and instead suggested a binocular projection focally in the visual field. To avoid distraction, it is important to minimize interference of augmented visual cues with normal visual perception. Therefore, visual cues should be displayed as if they are part of the environment, e.g., augmented bars (AB) that are displayed as if they are placed on the floor. This demands that the position and size of the augmented cues are updated in real time, depending on the position and orientation of the head and the walking speed. Also, it requires the smart glasses to have a sufficiently wide field of view. In addition, to enable users to adjust their steps to augmented visual cues, the augmented cues should start close to the user’s body. Furthermore, previous reports (16, 17) suggested that three-dimensional (3D) cues might be more effective in reducing FOG than two-dimensional cues, as were used in previous studies (12, 15). Equally spaced transverse bars on the floor as well as a staircase, either real or as painted optical illusion (17), can constitute such 3D cues. Whether the presentation of transverse bars and staircases *via* AR influences FOG and gait still needs to be explored. However, smart glasses with displays that are binocular (to enable 3D cues), tiltable and with a sufficient field of view (to allow for display of the cues close to the user), are not yet commercially available. For this purpose, we developed a prototype of custom-made smart glasses and software to provide 3D transverse bars or a staircase in AR. For augmented visual cues to be useful, they should be at least as effective as commonly applied cueing strategies such as conventional 3D transverse bars on the floor (16) or auditory cueing *via* a metronome (18). It should be carefully investigated whether wearing smart glasses, even when switched off, interferes with the effects of external cues. Possibly, augmented visual cues might not only affect FOG provoked by spatially demanding situations such as gait initiation but also those provoked by temporal triggers such as turning while walking. Originally, visual cues were thought to provide spatial information that could aid patients in scaling their movements (18), while auditory cues are considered to provide an external rhythm to which movements can be coupled to in the presence of a disrupted internal rhythm (18–20). Interestingly, moving visual targets have also been shown to improve motor timing in finger tapping tasks in healthy individuals, thereby activating regions in the basal ganglia which are associated with motor control and temporal processing (21, 22). Whether moving visual cues, such as augmented visual cues updated in real time, can reduce both spatially and temporally triggered FOG is not yet known. In addition to their effectiveness, user satisfaction should be carefully investigated to assess the usability of 3D augmented visual cues delivered by smart glasses.

The present study investigates the usability of 3D augmented visual cues delivered by smart glasses in comparison to conventional 3D transverse bars on the floor and auditory cueing *via* a metronome in reducing the occurrence of FOG, the percentage of time spent on FOG, and the variability of stride length and cycle time.

## Materials and Methods

### Participant Selection

This study was performed in accordance with the guidelines of the Declaration of Helsinki (1964) and was approved by the medical ethics committee Twente. All subjects provided written informed consent prior to their inclusion in the study. Persons aged over 18 years, with PD according to the UK Brain Bank criteria (23), and subjective presence of FOG [score 1 on question 1 from the New Freezing of Gait Questionnaire (NFOGQ) (24)] more than once per day (score 3 on question 2 from the NFOGQ) were eligible for inclusion. Exclusion criteria were stroke in the medical history, psychiatric disease interfering with assessment of FOG, severe uncorrected visual or hearing impairments disabling the participant to perceive visual or auditory cues, comorbidity limiting ambulation, inability to walk unaided, a deep brain stimulator or apomorphine pump, jejunal levodopa gel infusion, and severe cognitive impairments [mini-mental state examination (MMSE) <24 at the moment of inclusion]. As in several previous studies (12, 25), participants were tested at the end of their regular dopaminergic medication cycle (i.e., while experiencing the end-of-dose phenomenon), because this closely resembles the real-life situation where the most FOG occurs during an OFF state when the dopaminergic medication has been wearing off. Thus, participants were tested at the time when they would normally take their (after-)noon levodopa and were instructed to postpone this levodopa intake until after the walking trials. Prior to testing, the following questionnaires were taken: NFOGQ (24), MMSE (26, 27), frontal assessment battery (28), and Movement Disorder Society Unified Parkinson’s Disease Rating Scale (MDS-UPDRS) part III (29).

### Smart Glasses System

**Figure 1:**
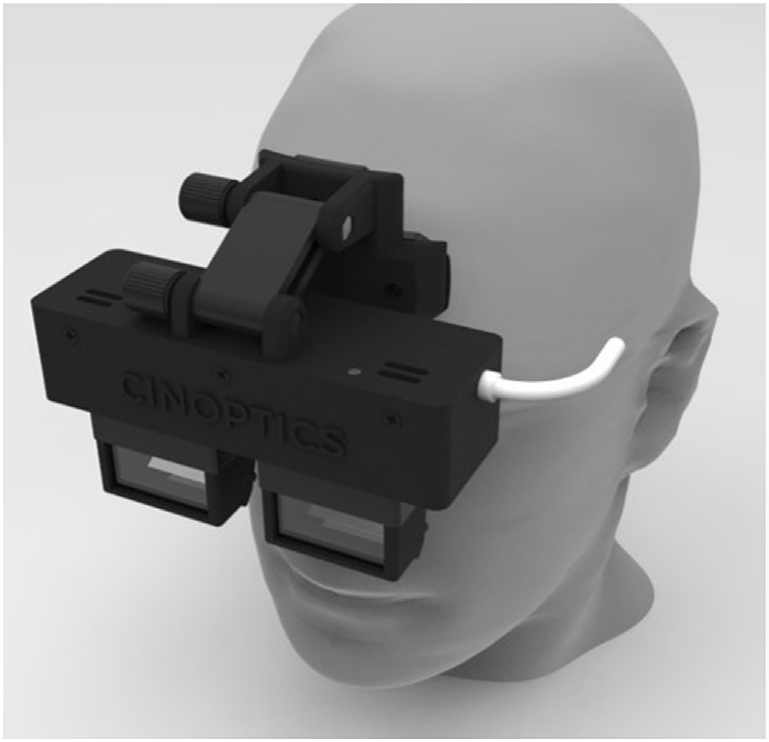
Smart glasses. Illustration of the prototype of custom-made smart glasses (Cinoptics, Maastricht, the Netherlands) on a model. The prototype is specifically designed for a large field of view and adjustable angle to allow augmented reality visual cues to be presented as if they are placed on the floor. Binocular see-through displays are mounted in a black frame attached to adjustable head straps (not shown here).

A prototype of custom-made smart glasses (Cinoptics, Maastricht, the Netherlands) was used to display the augmented cues and was worn throughout the experiment (Figure 1). These binocular smart glasses contained two CE-certified see-through optical color displays (organic light-emitting diodes, with 1,280 × 720 pixel resolution, a 60 Hz refresh rate, and a diagonal field of view of 45°), which could be tilted up to 30°. The participant’s head orientation was measured with an inertial measurement unit (IMU) with a sampling frequency of 160 Hz. The displays were mounted in a black frame attached to adjustable head straps, weighting 530 g altogether. The smart glasses were connected with a Microsoft Surface Pro 4 tablet carried inside a mesh pack worn on the participant’s back. In addition, participants wore an MVN Awinda motion capture system (Xsens, Enschede, the Netherlands) for collection of motion data. This system consisted of 17 IMUs with 3D gyroscopes, accelerometers, and magnetometers (60 Hz sampling frequency, 30 ms latency) attached to the feet (2), lower legs (2), upper legs (2), pelvis (1), hands (2), forearms (2), upper arms (2), sternum (1), shoulders (2), and head (1) with Velcro straps. The sensors were calibrated without the participant wearing the smart glasses (to avoid magnetic disruption of orientation) at the start of the experiment and recalibrated during the experiment if the sensor orientation was disrupted. The motion data were transmitted *via* a wireless local area network to a laptop with the MVN studio 4.2.0 software installed, and then to the tablet. Custom-made software on the tablet used the incoming data from the IMUs of the smart glasses and the motion capture system to update the position of the augmented cues displayed by the smart glasses in real time. This resulted in the augmented cues being displayed as if they were placed on the floor.

### Cues

The following five conditions were tested: 3D augmented transverse bars (AB) (see Video S1 in Supplementary Material for an illustration), 3D augmented staircase (AS) (Video S2 in Supplementary Material), equally spaced transverse conventional bars (CB) on the floor, auditory cueing *via* a conventional metronome (CM) and no cues (OFF). The smart glasses were worn during all conditions. The dimensions of the AB were set to match those of the CB, which measured 914 mm (width) × 19 mm (depth) × 19 mm (height) with a distance in between the bars of 40% of the participant’s height rounded to the nearest 5 cm, based on previous studies (9, 15, 30). The AS was set to match a real staircase measuring 914 mm (width) × 254 mm (depth) × 196 mm (height). The position of the AB and staircase was adjusted in real time according to the walking speed and head orientation of the participant. The bars in conditions CB and AB and the staircase in condition AS were all colored white. The metronome in condition CM was played *via* speakers at a clearly audible volume, at 110% of a participant’s preferred cadence (25, 31–33).

### Walking Courses

**Figure 2:**
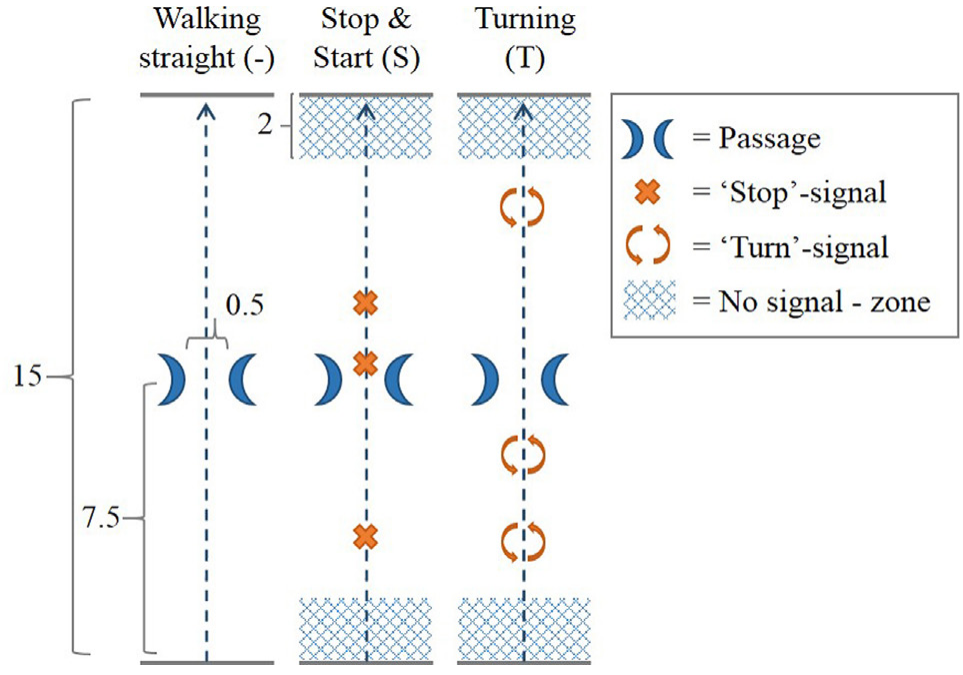
Walking courses. In each of three walking courses, the participant walked across a 15 m long walking trajectory with a passage at the middle of the trajectory created by two chairs 0.5 m apart. In the walking straight (−) course, no additional task was performed. In the “stop and start” (S) course, prerecorded voice commands signaled the participants to stop walking at three random distances along the track. Participants were instructed to resume walking on their own initiative. In the “turning” (T) course, participants were signaled by prerecorded voice commands to make a full 360° turn at three random distances along the track. No stop-signals or turn-signals were given in the “no signal—zone” at the first and last 2 m of the walking trajectory. All measures in Figure 2 are given in meters.

The walking trajectory consisted of a 15 m walking track along an empty corridor at the University of Twente, with a passage at 7.5 m made-up by two chairs placed 50 cm apart (Figure 2). Three different walking courses were performed along this walking trajectory. In the “walking straight” (−) course, participants walked along the walking trajectory without any additional task. In the “stop and start” (S) course, prerecorded voice commands signaled the participants to stop walking at three random distances along the track; they were instructed beforehand to resume walking on their own initiative. In the “turning” (T) course, participants were signaled by prerecorded voice commands to make a full 360° turn at three random distances along the track. No stop-signals or turn-signals were given in the “no signal—zone” at the first and last 2 m of the walking trajectory.

The experiment consisted of two sessions separated by a half an hour break. Each session consisted of five “blocks” with one condition (AB, AS, CB, CM, or OFF) per block. A block included all the walking courses (−, S, T) performed once. Hence, each condition–course combination was performed once per session. In between blocks, participants were offered to rest as long as needed. The order of the conditions (AB, AS, CB, CM, OFF), the courses (−, S, T) and the timing of the “stop” and “turn” signals were balanced by the experiment control software on the laptop. Experiments were performed in a single visit, lasting on average 2.5–3 h.

Prior to the experiment, participants familiarized themselves with the smart glasses, the augmented cues, and the CB. Each walking course was explained, shown, and practiced until performed correctly. Participants were not instructed in explicit detail on how to handle the cues. After the first session, participants were asked whether they wanted to continue with the second session after the break, or quit (for example, because of tiredness).

### User Interview

A semi-open structured interview was performed after the walking trials to assess participants’ experience with the smart glasses and cues (Table S1 in Supplementary Material). This interview encompassed questions and statements regarding the use of technical devices, usefulness of the four different cues (AB, AS, CB, and CM), ease of use and learning, satisfaction with the glasses and cues, preferences, and suggestions regarding the glasses and cues. Participants were asked to rate on a 5-point Likert scale (1 representing “totally disagree,” 5 “totally agree”) how much they agreed with the statements and were invited to elaborate on their answer. For question 7, which asked for cueing condition preferences, the condition preferred the most (question 7.1) was assigned 5 “preference points,” the second most preferred condition (question 7.2) 4 preference points, and so on up to the least preferred condition (question 7.5). Preference points were summed per condition.

### Data Analysis

A video recorder at each end of the walking trajectory recorded all trials on video for *post hoc* analysis of FOG. In accordance with the current working definition, FOG was defined as “brief, episodic absence or marked reduction of forward progression of the feet despite the intention to walk” (1). Two independent raters (Sabine Janssen and Jorik Nonnekes) were blinded for the condition (except for the CB) and scored the videos for number and duration of FOG per trial. Discrepant ratings were discussed until consensus was reached. Motion data from the Awinda motion capture system were wirelessly transmitted to MVN studio version 4.2.0. Orientation and position data, calculated by MVN studio, together with raw accelerometer and gyroscope data were exported to Matlab R2014b (Mathworks, Inc., Natick, MA, USA) for the offline calculation of gait parameters as previously described (12).

Primary performance measures were the number of FOG episodes, the percentage of time spent on FOG, and the variability (represented by the SD) of the stride length and cycle time. Secondary performance measures were the stride length, cycle time, cadence, and speed.

All statistical tests were performed in IBM SPSS version 24. An alpha of 0.05 was applied for all two-sided tests. Normality of distributions was tested with the Shapiro–Wilk test. Central tendency and statistical dispersion are given as the mean and SD if distributions were normally distributed, and otherwise as the median and interquartile range. The number of FOG episodes and the percentage of time spent on FOG (calculated as the cumulative duration of FOG divided by the summed duration of trials multiplied with 100, per individual and per condition) were compared in participants who experienced at least one FOG episode throughout the experiment. Sub-analyses were performed for FOG episodes occurring during turning and during non-turning events. In addition, sub-analyses were performed for the number of FOG episodes and the percentage of time spent on FOG in the participants who experienced the most FOG episodes (defined as a total number of FOG episodes above the median number of FOG episodes in all participants with at least one FOG episode).

The mean and SD of the step length and of the time to complete one gait cycle (cycle time), cadence, and walking speed were analyzed exclusively for the “walking straight” courses, in all participants. Kinematic parameters were calculated as the median values per participant, per condition, and then compared across participants for each cueing condition. A one-way repeated measures ANOVA was applied in the case of normally distributed data. If the assumption of sphericity, as assessed by Mauchly’s test of sphericity, was violated, a Greenhouse–Geisser correction was applied. The non-parametric Friedman test was used in case of non-normality. All *post hoc* pairwise comparisons were performed with a Bonferroni correction for multiple comparisons.

From the exit interview, median scores are reported for questions answered on a 5-point Likert scale. Questions with open answers and elaborations on the closed questions were qualitatively assessed.

## Results

Clinical characteristics of the participants are summarized in Table 1. All 25 participants completed the first session. Five participants did not perform the second session because of physical tiredness, resulting in 20 participants who completed both sessions. The results of the statistical tests on FOG and gait parameters are summarized in Table 2.

**Table 1:** Clinical characteristics of the participants (*N* = 25).

	Median	Range
Age (years)	72	65–79
Gender (% male)	76	
Height (cm)	171	159–189
Body mass index (kg/m^2^)	27.1	21.7–37.2
Disease duration (years)	11	3–20
Years since FOG (years)	2	0.25–12
Daily levodopa dosage (mg/day)	750	0–1,200
UPDRS-part III	34	10–61
UPDRS-PIGD	6	2–12
Hoehn and Yahr	2	2–3
MMSE	28	19–30
NFOGQ	18	8–28
FAB	14	5–26

*FOG, freezing of gait; UPDRS-part III, Unified Parkinson’s Disease Rating Scale part III: motor examination; UPDRS-PIGD, Unified Parkinson’s Disease Rating Scale—postural instability and gait disorder (question 3.9 up to 3.13 from UPDRS-part III); MMSE, mini-mental state examination; NFOGQ, New Freezing of Gait Questionnaire; FAB, frontal assessment battery*.

*All questionnaires, including the UPDRS, were rated while participants were end-of-dose*.

**Table 2:** FOG and gait parameters per condition.

Parameter	Condition					*p*-Value
	OFF	CB	CM	AB	AS	
**FOG parameters^a^**						
Mean number of FOG per trial	0.08 (0.11)	0.10 (0.08)	0.09 (0.14)	0.11 (0.19)	0.13 (0.15)	0.042^†A^
% Time spent on FOG	9.05 (12.11)	12.73 (13.08)	12.34 (16.86)	12.41 (15.28)	15.56 (13.68)	0.090^†^
**Gait parameters^b^**						
Stride length variability	0.17 (0.12)	**0.21 (0.10)^B^**	0.17 (0.13)	0.16 (0.14)	**0.15 (0.07)^B^**	0.001*
Cycle time variability	0.24 (0.06)	0.31 (0.27)	0.24 (0.12)	0.24 (0.12)	0.21 (0.13)	0.117*
Stride length (m)	0.92 (0.35)	**1.19 (0.57)^C,D^**	0.94 (0.37)	**0.93 (0.32)^C^**	**0.86 (0.37)^D^**	0.001*
Cycle time (s)	**1.15 (0.16)^E^**	**1.60 (0.33)^E,F,G,H^**	**1.15 (0.16)^F^**	**1.18 (0.25)^G^**	**1.18 (0.16)^H^**	<0.0005^†^
Cadence (steps/min)	**102.76 (13.88)^I^**	**74.41 (15.83)^I,J,K,L^**	**102.81 (14.38)^J^**	**100.40 (19.63)^K^**	**99.85 (13.41)^L^**	<0.0005^†^
Speed (m/s)	**0.83 (0.41)^M^**	**0.72 (0.44)^M,N^**	**0.84 (0.42)^N^**	0.78 (0.34)	0.75 (0.40)	0.001^†^

*^a^FOG parameters in mean (SD), in participants with more than one FOG episode throughout the experiment (*N* = 19); all walking courses*.

*^b^Gait parameters in median (interquartile range), in all participants (*N* = 25); during “straight-walking” courses*.

*FOG, freezing of gait; OFF, smart glasses switched OFF; CB, conventional bars; CM, conventional metronome; AB, augmented bars; AS, augmented staircase*.

p-Values for within group differences are calculated with a one-way repeated measures ANOVA (*) or Friedman test (^†^). Statistically significant differences (adjusted p < 0.05, two-sided tests) between pairs of cues are printed bold, with the test statistic and p-value given in the legend. ^A^Upon post hoc pairwise comparisons no statistically significant differences between cue-pairs. ^B^χ^2^ 2.048, p < 0.0005. ^C^χ^2^ 1.571, p = 0.013. ^D^χ^2^ 1.762, p = 0.003. ^E^p < 0.0005, 95% CI difference CB–OFF 0.30–0.55. ^F^p < 0.0005, 95% CI difference CB–CM 0.31–0.54. ^G^p < 0.0005, 95% CI difference CB–AB 0.26– 0.49. ^H^p < 0.0005, 95% CI difference CB–AS 0.27–0.52. ^I^p < 0.0005, 95% CI difference CB–OFF −32.42 to −18.56. ^J^p < 0.0005, 95% CI difference CB–CM −31.88 to −20.07. ^K^p < 0.0005, 95% CI difference CB–AB −30.09 to −15.19. ^L^p < 0.0005, 95% CI difference CB–AS −30.99 to −17.02. ^M^p = 0.019, 95% CI difference CB–OFF −0.23 to −0.02. ^N^p = 0.007, 95% CI difference CB–CM −0.22 to −0.03.

### Freezing of Gait

**Figure 3:**
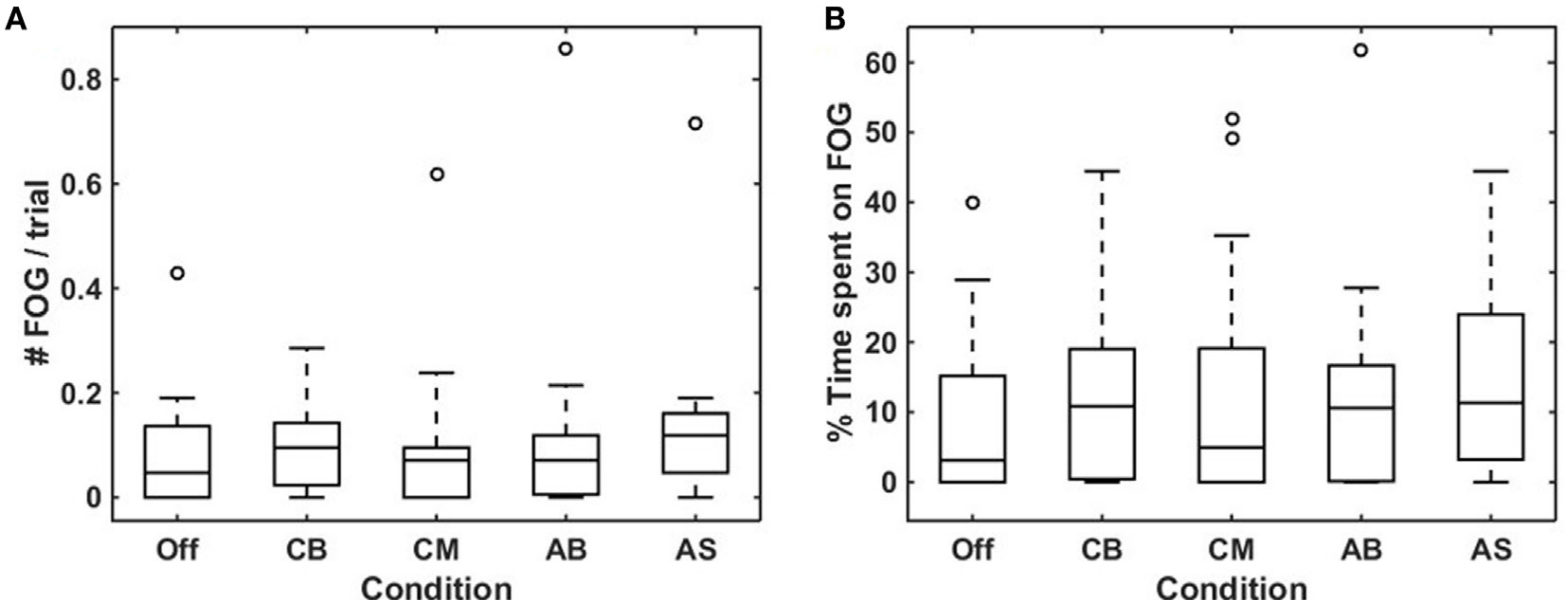
Effects of conditions on freezing of gait (FOG) occurrence. Boxplots visualizing the effect of the five conditions on mean number of FOG episodes per trial **(A)** and percentage of time spent on FOG **(B)** for each condition in participants who experienced more than one FOG episode throughout the experiment (*N* = 19). Off, smart glasses worn but switched off; CB, conventional bars; CM, conventional metronome; AB, augmented bars; AS, augmented staircase.

There was a high degree of consensus between raters (Sabine Janssen and Jorik Nonnekes) on the rating of number [*r_s_*(23) = 0.979, *p* < 0.0005] and total duration of FOG episodes [*r_s_*(23) = 0.974, *p* < 0.0005] per participant. In 19 out of 25 participants, at least one FOG episode occurred during the experiment, with a total of 300 FOG episodes for all persons together. Of these, 18 participants experienced a total of 224 FOG episodes during turning, and 8 participants experienced FOG during walking straight (20 episodes), gait initiation (18 episodes), passing the passage (21 episodes), or upon coming to a standstill (17 episodes). Only participants in whom at least one FOG episode occurred (*N* = 19) were included in the analysis of the effect of cues on FOG. The number of FOG episodes (Figure 3A) and the percentage of time spent on FOG (Figure 3B) were non-normally distributed, hence the Friedman test was used. Although there was a statistically significant difference amongst the various cues for number of FOG episodes, pairwise comparisons failed to show a significant difference. There was no statistically significant difference in the percentage of time spent on FOG amongst the different cues. Results were similar when performing a sub-analysis for FOG episodes occurring during turning (representing temporally triggered FOG) and during non-turning events (representing spatially triggered FOG). Sub-analyses among participants with the greatest number of FOG episodes (*N* = 10) again showed no statistically significant difference in number of FOG episodes nor in the percentage of time spent on FOG across the five conditions.

### Gait Variability

**Figure 4:**
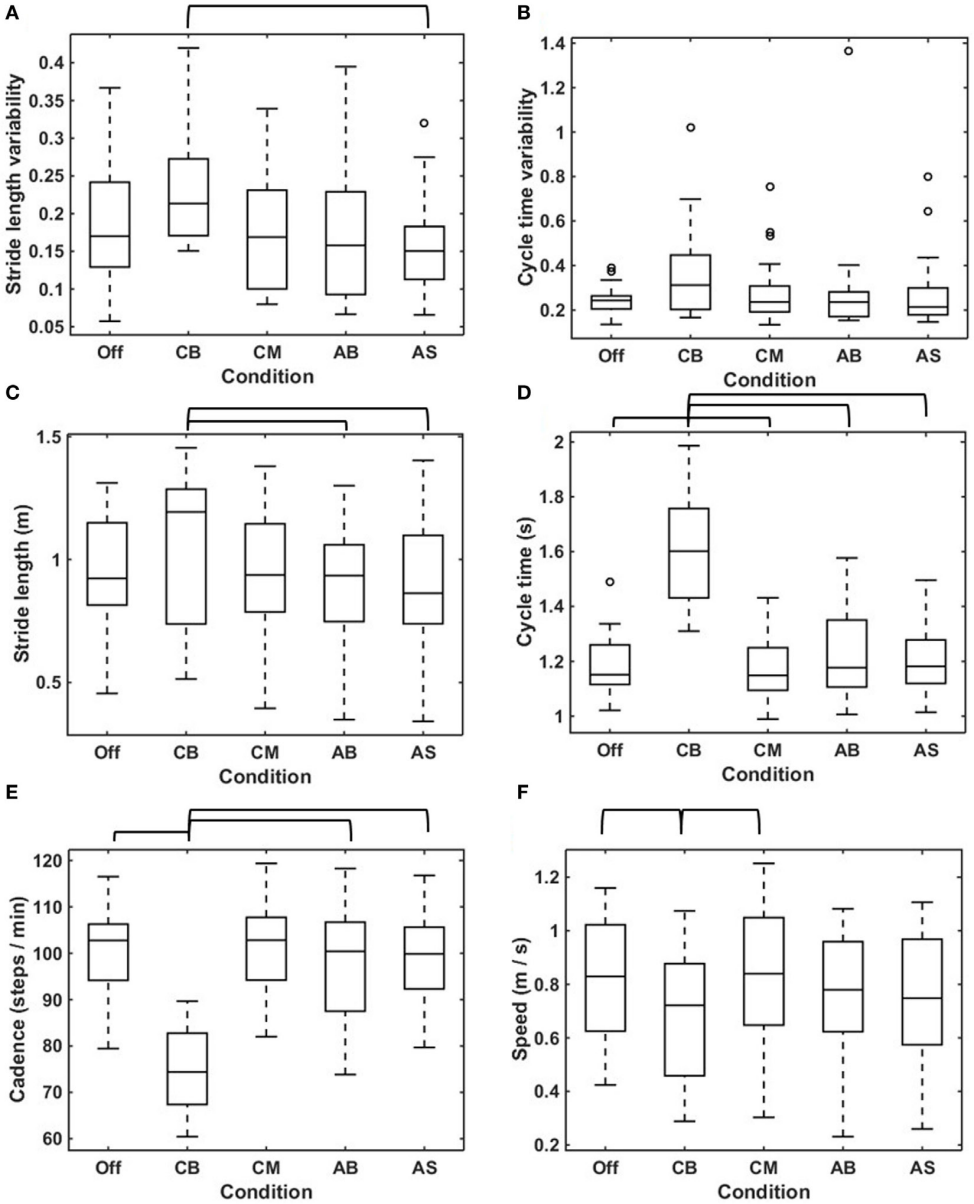
Effects of conditions on gait parameters. Boxplots visualizing the effects of the five conditions on stride length variability **(A)**, cycle time variability **(B)**, mean stride length **(C)**, mean cycle time **(D)**, cadence **(E)**, and speed **(F)** calculated in straight-walking trials in all participants (*N* = 25). The brackets indicate statistically significant differences in the parameters concerned between conditions (*p* < 0.05). The statistical test values are given in Table 2.

The median stride length variability was statistically significant higher for the CB compared to the AS (Figure 4A; Table 2). There was no statistically significant difference in cycle time variability amongst the various conditions (Figure 4B; Table 2).

### Stride Length and Cycle Time

The stride length was statistically significant larger for the conservative bars compared to the AB and the AS (Figure 4C; Table 2). The median cycle time showed one outlier for the no cue condition; exclusion of the outlier did not change the results, hence the outlier was included in the analysis. The assumption of sphericity was violated [χ^2^(9) = 28.564, *p* = 0.001], and therefore a Greenhouse–Geisser correction was applied (ε = 0.555). The median cycle time was statistically significant higher for the CB when compared to the no cue condition, the CM, the AB, and the AS (Figure 4D; Table 2).

### Cadence and Speed

Cadence showed no outliers, and the assumption of sphericity was not violated [χ^2^(9) = 13.979, *p* = 0.124]. The median cadence was lower for the CB compared to the no cue condition, the CM, the AB, and the AS (Figure 4E; Table 2). For speed, the assumption of sphericity was violated [χ^2^(9) = 24,325, *p* = 0.004], hence a Greenhouse–Geisser correction was applied (ε = 0.594). The median speed was lower for the CB compared with the no cue condition and the CM (Figure 4F; Table 2).

### User Experience

Overall, the CM was preferred the most (99 preference points), followed by the CB (80 preference points), AB (77 preference points), no cues (61 preference points), and AS (58 preference points). Participants indicated they could walk better with cues (AB/AS/CB/CM 4), that all cues except the AS made walking easier (AS 3; AB/CB/CM 4), and that they considered all cues except the AB useful (AB 3; AS/CB/CM 4). The metronome was considered the most well-suited cue to provide more control over daily life activities (CM 4; AB/AS/CB 3) and met participants’ needs (CM 4; AB 2; AS/CB 3) and expectations (CM 4; AB/AS 3; CB 2) the most. The AB and CB were considered less distracting than the AS and CM (AB/CB 4; AS/CM 3). Ease of use, usability, and willingness to use the smart glasses in everyday life were rated low (2). The use of smart glasses did not require additional effort (3), and walking with smart glasses was considered easy to learn (4). Participants suggested to improve the comfort, esthetics, usability, field of view, and stability of the smart glasses on the head and to reduce their weight and size. With regard to the augmented cues, three participants suggested to experiment with softer colors than the current white, and three participants suggested to broaden the augmented cues, and one participant wished for footsteps on the AS.

## Discussion

The present study investigated the usability of 3D augmented visual cues delivered by smart glasses, conventional 3D bars on the floor, a metronome or no cues on the occurrence of FOG, the percentage of time spent on FOG, the (variability of) stride length and cycle time, cadence, and speed. Note that the smart glasses were worn during all conditions, but only switched on for the AB and staircase. Neither the AB and staircase, the CB on the floor, nor the metronome reduced the number of FOG episodes or the percentage of time spent on FOG. Results were similar in the subset of FOG episodes occurring during spatially demanding situations, when the FOG episodes triggered by turning were excluded. The CB caused an increase in stride length, cycle time and stride length variability, and a decrease in cadence and speed. There was no effect of the other cues on gait parameters.

That the CB on the floor and the metronome failed to reduce FOG contradicts studies reporting a reduction in FOG by visual (16, 34) or auditory (34, 35) cues. The influences of CB on gait parameters could be attributable to the distance between the bars depending on the participant’s height (leading to larger and slower steps if the distances between the bars were larger than a participants’ preferred uncued step length), and the observation that participants varied the number of steps in between two bars, increasing stride length variability. That other cues did not alter gait parameters does not correspond to earlier studies (7, 10, 11).

We propose several possible explanations for the lack of effects of cues on FOG and gait parameters. First, participants were not used to walking with smart glasses, and this novel experience, together with their experience of the smart glasses being quite heavy and uncomfortable, might have caused distractions. It is well recognized that dividing attention is impaired in PD-FOG (36), and FOG severity has been correlated with difficulties in switching attention (37). Dual tasks, which also require switching or dividing attention, are known to deteriorate FOG (38) and to counteract the FOG-alleviating effects of visual cues (39). Considering that FOG occurrence did not differ amongst conditions (including with the smart glasses switched off), the smart glasses themselves rather than the cues might have caused distraction. This may have canceled out the FOG-ameliorating effect of cues. With regard to gait parameters, dual tasks are known to decrease step length, walking speed (39), and increase cadence (38) and step length variability (39) in PD-FOG, effects which are undone in the presence of visual cues (39). Because a condition without smart glasses was not included, we cannot rule out that the smart glasses induced distraction, similar to a dual task, altering these gait parameters. However, the previous observation that dual task-induced gait alterations could be reversed by visual cues (39) was not found in our study. The rather “bulky” design of this prototype of smart glasses was due to technical constraints raised by the requirements to deliver 3D augmented cues as if placed in the real environment. Second, the duration of the experiment might have been insufficient for participants to familiarize themselves with the smart glasses and cues. Indeed, in former studies, immediate effects of cues were variable, while longer periods of cueing training were thought to be more effective (18). Third, the frame of the smart glasses blocked part of the peripheral visual field. This might have reduced the visual feedback which persons with PD-FOG are more reliant on due to impaired propriocepsis (40–43). A previous study showed that blocking the view of the lower limbs caused an increase in FOG, which visual cues did not prevent (39). Hence, the frame of the smart glasses might have reduced visual sensory feedback, thereby increasing FOG occurrence in all conditions, which was not reversed by visual cues. In addition, blockage of the visual field has previously shown to decrease step length, velocity, and cadence, which was reversible with visual cues in one (39), but not in another study (41). Such difference in gait parameters between visually cued and un-cued conditions could not be confirmed in our study. Fourth, the augmented visual cues as well as the CB were all perceived in the central visual field. It has been suggested that the integration of information from the central and peripheral visual fields is important for the perception of self-movement (44) and that typically a stationary center with a moving periphery induces a sense of self-movement. Moving visual cues in the central visual field, such as in our experiment, constitute the opposite situation. This might influence the sense of self-motion, thereby affecting motor planning and potentially contributing to the occurrence of FOG (45). However, currently used visual cues such as bars on the floor or laser lights (46–48) are predominantly presented in the central visual field, while an enhanced peripheral optic flow delivered *via* Google Glass did not reduce FOG (12). Fifth, dopaminergic medication levels at the end-of-dose might have interfered with the effects of cueing. Studies finding no effects of cues on FOG and gait parameters were predominantly performed in the ON state (15, 49–52). However, rather than that medication interferes with the effects of cues, these studies might have been underpowered to find effects of cues on gait parameters that (due to the symptomatic effect of medication) were less severely disturbed than in the OFF state. Positive effects of cues have been reported by studies performed in the OFF (15, 34, 35, 52, 53), ON (6, 10) as well as the end-of-dose state (25). The role of medication state on response to cues remains to be established.

A limitation of this study is the absence of a control condition without smart glasses and cues, which would have allowed to distinguish distraction by the smart glasses, as discussed above. Furthermore, 224 out of 300 FOG episodes occurred during turning, which might be more receptive to temporal than spatial cues. The remaining 76 non-turn FOG episodes, which could potentially be more sensitive to visual cues, might have been too few to find a statistically significant effect.

In conclusion, 3D augmented visual cues delivered by customized smart glasses did not improve FOG nor gait stability in persons with PD-FOG. Adjustments to smart glasses are prerequisite to turn them into effective cueing devices, amongst others by a more lightweight, comfortable, and user friendly design, a wider field of view and less interference with sensory visual feedback. Future research should investigate whether, and through which mechanisms, 3D cues are more effective than 2D cues; whether novel cues affect FOG provoked by spatial as well as temporal triggers; and whether visual cues should be presented in the central or peripheral visual field. Furthermore, it is of particular interest whether a larger effect of augmented visual cues can be obtained with a longer habituation period, or when cues are provided “on demand.” Ideally, future studies should include healthy control individuals to assess whether cues affect gait parameters differently in persons with PD and healthy controls. To avoid a “trial-and-error”-based development of new cueing devices, it is important to deepen our insights into the characteristics of effective cues, requirements for new cueing devices, and the neuronal mechanisms underlying externally cued (freezing of) gait.

## Ethics Statement

This study was performed in accordance with the guidelines of the Declaration of Helsinki (1964) and was approved by the medical ethics committee Twente. All subjects provided written informed consent prior to their inclusion in the study.

## Author Contributions

SJ contributed to the study design and to the acquisition, analysis, and interpretation of the data, drafted the manuscript, and edited the final manuscript for submission. BB contributed to the concept and design of the study, to the development of the software investigated in the study, and to the critical appraisal of the manuscript. JN was involved in the conception and design of the current work, in the acquisition and interpretation of data and critically revised the manuscript. MB was involved in the acquisition of the data and critically appraised the manuscript. BRB contributed to the study design and revision of the manuscript. TH, YZ, and RW were involved in the conceptual design and setup of this study, in the interpretation of the data, and in the critical revision of the manuscript. All the authors gave their approval of the final manuscript.

## Conflict of Interest Statement

The author declares that the research was conducted in the absence of any commercial or financial relationships that could be construed as a potential conflict of interest. The reviewer, DR-M, and handling editor declared their shared affiliation and the handling editor states that the process nevertheless met the standards of a fair and objective review.

## Supplementary Material

Video S1 Illustration of the simulation of three-dimensional (3D) transverse bars displayed in augmented reality (AR) perceived through the smart glasses. The upper half of the screen represents a top view of the walking direction of the user; here walking forward, turning 90° to the left and resuming to walk forward. The lower half of the screen represents the AR display. White 3D transverse bars are updated in real time upon movement of the user. The black area represents the part of the display where no AR images are being displayed, and the “real environment” is transmitted to be perceived by the user.

Video S2 Illustration of the simulation of a three-dimensional (3D) staircase displayed in augmented reality (AR) perceived through the smart glasses. The upper half of the screen represents a top view of the walking direction of the user; here walking forward, turning 90° to the left and resuming to walk forward. The lower half of the screen represents the AR display. A white 3D staircase is updated in real time upon movement of the user. The black area represents the part of the display where no AR images are being displayed, and the “real environment” is transmitted to be perceived by the user.

## Abbreviations

PD, Parkinson’s disease; FOG, freezing of gait; OFF, smart glasses switched OFF; CB, conventional bars; CM, conventional metronome; AB, augmented bars; AS, augmented staircase.
