# Supplementary material for: Usability of Three-dimensional Augmented Visual Cues Delivered by Smart Glasses on (Freezing of) Gait in Parkinson’s Disease
Source: Front Neurol. 2017 Jun 13;8:279. doi: 10.3389/fneur.2017.00279 (PMC5468397; doi:10.3389/fneur.2017.00279)
Supplement: Supplementary file 1 [file Table_1.DOCX]

## Supplementary table 1 User interview questions and statements

| **1** | **Medical history** | |
| --- | --- | --- |
| 1.1 | “When did you first experience symptoms of Parkinson’s disease?” | Open |
| 1.2 | “Which were those first symptoms?” | Open |
| 1.3 | “When did you develop freezing of gait?” | Open |
| 1.4 | “Do you use cues to reduce freezing of gait?” | Yes / No |
| 1.5 | If ‘yes’ to question 1.4: “which cues?” | Open |
| **2** | **Use of technical devices** | |
| 2.1 | “Do you use a mobile phone (not a smartphone)?” | Yes / No |
| 2.2 | “Do you use a smartphone?” | Yes / No |
| 2.3 | “Do you use a tablet or iPad?” | Yes / No |
| 2.4 | “Do you use a computer or laptop?” | Yes / No |
| **3** | **Usefulness of cues** | |
| 3.1 | “The augmented bars via the smart glasses […] | |
| 3.1.1 | […] improved my walking.” | Likert scale |
| 3.1.2 | […] are useful.” | Likert scale |
| 3.1.3 | […] could give me more control over daily life activities.” | Likert scale |
| 3.1.4 | […] make walking easier.” | Likert scale |
| 3.1.5 | […] fulfill my needs.” | Likert scale |
| 3.1.6 | […] do everything I expected it to do.” | Likert scale |
| 3.1.7 | […] are NOT distracting.” | Likert scale |
| 3.2 | “The bars on the floor […] | |
| 3.2.1 | […] improved my walking.” | Likert scale |
| 3.2.2 | […] are useful.” | Likert scale |
| 3.2.3 | […] could give me more control over daily life activities.” | Likert scale |
| 3.2.4 | […] make walking easier.” | Likert scale |
| 3.2.5 | […] fulfill my needs.” | Likert scale |
| 3.2.6 | […] do everything I expected it to do.” | Likert scale |
| 3.2.7 | […] are NOT distracting.” | Likert scale |
| 3.3 | “The augmented staircase via the smart glasses […] | |
| 3.3.1 | […] improved my walking.” | Likert scale |
| 3.3.2 | […] are useful.” | Likert scale |
| 3.3.3 | […] could give me more control over daily life activities.” | Likert scale |
| 3.3.4 | […] make walking easier.” | Likert scale |
| 3.3.5 | […] fulfill my needs.” | Likert scale |
| 3.3.6 | […] do everything I expected it to do.” | Likert scale |
| 3.3.7 | […] are NOT distracting.” | Likert scale |
| 3.4 | “The metronome […] | |
| 3.4.1 | […] improved my walking.” | Likert scale |
| 3.4.2 | […] is useful.” | Likert scale |
| 3.4.3 | […] could give me more control over daily life activities.” | Likert scale |
| 3.4.4 | […] makes walking easier.” | Likert scale |
| 3.4.5 | […] fulfills my needs.” | Likert scale |
| 3.4.6 | […] does everything I expected it to do.” | Likert scale |
| 3.4.7 | […] was NOT distracting.” | Likert scale |
| **4.1** | **Ease of use** | |
| 4.1 | “The smart glasses are easy.” | Likert scale |
| 4.2 | “The smart glasses are user friendly.” | Likert scale |
| 4.3 | “Using the smart glasses requires little effort.” | Likert scale |
| 4.4 | “I would like to use the smart glasses regularly.” | Likert scale |
| 4.5a | “I would like to use the smart glasses on certain occasions.” | Likert scale |
| 4.5b | (if score ≥ 4 on question 4.5a) On what occasions? | Open |
| 4.6 | “The cues via the smart glasses were easily visible.” | Likert scale |
| **5** | **Ease of learning** | |
| 5.1 | “I learned to walk with the smart glasses quickly.” | Likert scale |
| 5.2 | “It is easy to learn to walk with the smart glasses.” | Likert scale |
| **6** | **Satisfaction** | |
| 6.1 | “I am satisfied with the smart glasses.” | Likert scale |
| 6.2 | “I would recommend the glasses to a friend.” | Likert scale |
| 6.3 | “It is fun to use the smart glasses.” | Likert scale |
| 6.4 | “The smart glasses work the way I want them to work.” | Likert scale |
| 6.5 | “The smart glasses are wonderful.” | Likert scale |
| 6.6 | “I feel that I need the smart glasses.” | Likert scale |
| 6.7 | “The smart glasses are pleasant to use.” | Likert scale |
| 6.8 | “If I could take the smart glasses home, I would do so.” | Likert scale |
| **7** | **Preferences** | |
| 7.1 | “You have walked with the smart glasses with the augmented bars and staircase, with the bars on the floor, with the metronome and without cues. Which cue did you prefer most?” | Likert scale |
| 7.2 | “Which cue did you prefer second?” | 1 – 5 |
| 7.3 | “Which cue did you prefer third?” | 1 – 5 |
| 7.4 | “Which cue did you prefer fourth?” | 1 – 5 |
| 7.5 | “Which cue did you prefer fifth, the least?” | 1 – 5 |
| 7.6 | “What adjustments to the augmented bars do you suggest?” | Open |
| 7.7 | “What adjustments to the augmented staircase do you suggest?” | Open |
| 7.8 | “What adjustments to the smart glasses do you suggest?” | Open |
| 7.9 | “Do you have remarks regarding the smart glasses or cues which have not yet been discussed?” | Open |

Questions and statements listed here are translated from Dutch. The Dutch questions and statements were read out to participants in a neutral voice. The last column lists the answer types. ‘Open’: verbalization of the answer without being offered a choice of answers. ‘Yes / No’: choice between ‘yes’ and ‘no’. ‘Likert scale’: agreement with the statement on a five-point Likert Scale, ranging from 1 (‘totally disagree’) to 5 (‘totally agree’); participants were welcomed to elaborate on their answer.
